# Measuring and modelling concurrency

**DOI:** 10.7448/IAS.16.1.17431

**Published:** 2013-02-12

**Authors:** Larry Sawers

**Affiliations:** Department of Economics, American University, Washington, DC, USA

**Keywords:** HIV, concurrency, sub-Saharan Africa, sexual network models, multiple concurrent partners

## Abstract

This article explores three critical topics discussed in the recent debate over concurrency (overlapping sexual partnerships): measurement of the prevalence of concurrency, mathematical modelling of concurrency and HIV epidemic dynamics, and measuring the correlation between HIV and concurrency. The focus of the article is the concurrency hypothesis – the proposition that presumed high prevalence of concurrency explains sub-Saharan Africa's exceptionally high HIV prevalence. Recent surveys using improved questionnaire design show reported concurrency ranging from 0.8% to 7.6% in the region. Even after adjusting for plausible levels of reporting errors, appropriately parameterized sexual network models of HIV epidemics do not generate sustainable epidemic trajectories (avoid epidemic extinction) at levels of concurrency found in recent surveys in sub-Saharan Africa. Efforts to support the concurrency hypothesis with a statistical correlation between HIV incidence and concurrency prevalence are not yet successful. Two decades of efforts to find evidence in support of the concurrency hypothesis have failed to build a convincing case.

This article addresses key issues in recent contributions to the literature over the role of concurrent heterosexual partnering in HIV epidemics in sub-Saharan Africa (SSA). In this literature, the term concurrency describes multiple partnering in which sexual relationships are overlapping rather than sequential. The focus of this article is the concurrency hypothesis, the assertion that unusually high levels of concurrency in SSA explain the region's exceptional epidemics of HIV. Empirical evidence and argumentation marshalled in support of the concurrency hypothesis have been examined closely and judged deficient, first by Deuchert [1] in 2007, then by Lurie and Rosenthal [2, 3] in 2009 and 2010 and subsequently by my own critiques co-authored with Eileen Stillwaggon and Alan Isaac [4, 5]. Supporters of the concurrency hypothesis have responded to these critics. (For example, see [6–10].) The objective of the present article is to re-examine the case for the concurrency hypothesis, incorporating information and insights drawn mostly from literature published after the earlier critiques, from recently published and unpublished data and from previously unpublished results of my own modelling.

Three critical issues raised in the recent debate over concurrency are explored in this article: measurement of the prevalence of concurrency, mathematical models of concurrency and HIV epidemic dynamics, and the correlation between HIV and concurrency. The article closes with a discussion of the research agenda and HIV-prevention policy that flow from that analysis.

Controversy over the hypothesis has mostly focused on two propositions underlying the hypothesis: concurrency is more prevalent in SSA than elsewhere and overlapping partnering is more effective than sequential partnering in spreading HIV [8, 11–13]. However, the two assertions are intertwined. It would weaken rather than strengthen the case in support of the concurrency hypothesis to show that concurrency could accelerate the spread of HIV, but only at levels exceeding those found in SSA. Thus, measuring and modelling concurrency are not two independent steps in determining the validity of the concurrency hypothesis but must be considered jointly.

## Measuring concurrency

Concurrency is straightforward conceptually (overlapping sexual partnering) but has been measured with various definitions that produce incomparable data of uneven quality. Modellers of sexual networks and HIV epidemic dynamics typically use measures of concurrency at a point in time (point prevalence) to describe the degree of concurrency in the modelled population, but published measures of concurrency are often the proportion of respondents who had a partnership overlap in the previous year or over their lifetime [4]. Nevertheless, point prevalence of concurrency is often not even half of one-year concurrency *in the same population*. Moreover, modellers often measure concurrency as a percentage of the entire modelled population. In contrast, survey researchers typically report concurrency as a percentage of sexually active or sexually experienced individuals [4]. The different denominators produce very different percentages used to describe the same level of concurrency. Finally, modellers typically present rates of concurrency for men and women together whereas survey data are almost always presented separately by gender. The differences in the way that modellers measure concurrency and the way that survey researchers measure concurrency has produced and continues to produce misunderstanding about what modelling says about HIV epidemics in SSA.

What follows presents recently published data on concurrency prevalence from surveys using the same measurement methodology and expressed the way modellers typically describe concurrency, as point prevalence for all adults. A discussion of types and plausible dimensions of error in measuring concurrency follows. The section ends by combining that information, showing a range of hypothetical rates of concurrency assuming plausible levels of reporting error.

### The UNAIDS protocol

In 2009, the Joint United Nations Programme on HIV/AIDS (UNAIDS) convened a panel of experts tasked with recommending a single method for measuring concurrency that avoided obvious problems with earlier questionnaire design [14]. The panel's recommendations are often described as the “UNAIDS guidelines” or the “UNAIDS protocol”, although UNAIDS did not formally endorse the proposal. The panel recommended asking respondents to specify dates of initial and most recent sexual contacts with their three most recent sexual partners in the previous year. Concurrency was to be measured at a point in time (point prevalence) six months prior to the interview as a percentage of all respondents aged 15–49. While disagreement over the best way to measure concurrency continues, the expert panel's recommendation focused the conversation in a useful way. In particular, the UNAIDS protocol has made it far easier to determine the external validity of modelling because it measures concurrency the same way that most modellers do.

Since 2009, 13 surveys have used the UNAIDS protocol in SSA. Two other surveys report concurrency for age groups different from those recommended by the UNAIDS expert panel, but otherwise followed the protocol (see Table 1). Eleven of the surveys are either Demographic and Health Surveys (DHS) or AIDS Indicator Surveys (AIS), which are nationally representative surveys conducted by national government statistical offices or ministries of health in collaboration with IFC International, an institution established by the US Agency for International Development. Four other surveys were in districts in countries in the region.

Most mathematical models of concurrency and HIV have the same number of men and women in the modelled population, so it is appropriate to present simple averages of men's and women's concurrency. (In every survey, women outnumbered men so the unweighted averages presented in Table 1 are higher than the weighted averages.) My colleagues and I have shown that gender asymmetry in concurrency does not have an important effect on modelled HIV epidemics, so the average of men's and women's concurrency appropriately describes a modelled population's degree of concurrency. (A. Isaac, E. Stillwaggon and L, Sawers, “Concurrency and the Spread of HIV: The Role of Gender Asymmetry,” Working Paper, American University, Washington, DC.) The unweighted average of men's and women's reported concurrency in the 11 countries and two districts with data for those aged 15–49 ranges from 0.8% to 7.6% and averages 3.4%.

**Table 1:** Point prevalence of concurrency measured using UNAIDS protocol in 15 surveys

	% of men	% of women	unweighted average	Source
**National Surveys for Ages 15–49**				
Burkina Faso	10.4	0.1	5.25	DHS 2011 [15]^1^
Burundi	1.5	0.0	0.77	DHS [16]^1^
Cameroon	13.3	1.9	7.60	DHS [17]
Ethiopia	2.3	0.0	1.15	DHS 2011 [18]
Lesotho	7.4	2.3	4.85	DHS 2009^2^
Malawi	3.8	0.1	1.95	DHS 2010 [19]
Mozambique	8.8	0.8	4.80	AIS 2009 [20]
Rwanda	1.5	0.1	0.80	DHS 2010 [21]
Senegal	5.1	0.2	2.65	DHS 2010–2011 [22]^1^
Uganda	9.7	0.4	5.05	DHS 2011 [23]^3^
Zimbabwe	3.8	0.3	2.05	DHS 2010–2011 [24]
**Sub-national Surveys for Ages 15–49**				
Uganda, rural district	9.8	0.4	5.10	Maher et al. [25]
S. Africa, Kwa-Zulu Natal	4.7	0.4	2.55	Eaton et al. [26]^4^
**Average for Ages 15–49**	6.3	0.5	3.43	
**Sub-National Surveys for Other Age Groups**				
Kenya, Kisumu, ages 18–24	4.0	3.5	3.75	Xu et al. [27]
Malawi, rural district, ages 15–59	12.0	–	–	Glynn et al. [28]^5^

1 The Burkina Faso 2011 DHS [15], Burundi 2011 DHS [16] and Senegal 2010–2011 DHS [22] report point prevalence only for men so women's concurrency is calculated using datasets from Measure DHS-IFC Macro (http://www.measuredhs.com/).

2 The official publication of the 2009 Lesotho DHS [29] does not report concurrency rates, so they are calculated using datasets from Measure DHS on which the DHS report is based.

3 The Uganda 2010 AIS [30] shows men's reported point prevalence of concurrency to be 4.5%, not the 9.7% reported in the Uganda 2011 DHS [23]. The two surveys were conducted by different agencies in Uganda. Both surveys state that they are nationally representative samples. Since the DHS and Maher *et al*. [25] report almost identical male concurrency, this table uses the higher figure even though the lower figure may be more reliable (the AIS had 4 times as many male respondents as the DHS). Adding further confusion, datasets from Measure DHS show that men's reported point prevalence in the Uganda DHS 2011 was 9.3%, not the published 9.7%.

4 Eaton *et al*. analyze men's concurrency, not women's, but add that “fewer than 0.4%” of women report concurrency [26].

5 Glynn *et al*. [28] report concurrency, using the UNAIDS protocol only for men age 15–59, but in Glynn *et al*.'s study and in Malawi as a whole ([30] Table 3.7), polygyny and thus concurrency is substantially higher among older men. Data for those aged 15–59 are thus not directly comparable to those in the age bracket specified in the UNAIDS protocol (15–49) [14]. Glynn *et al*. do not report women's concurrency measured with the UNAIDS protocol “since few women reported multiple partners”.

### New protocol produces data consistent with earlier measures

The rates of concurrency presented in Table 1 are similar to those found in earlier surveys in the region over the last 15 years. For example, a 2008 nationally representative survey in Zambia using a method similar to the UNAIDS protocol reported prevalence of concurrency at the time of interview among adults to be 4.2% [31]. Other evidence comes from 8 pre-2009 DHS in SSA, which report average one-year concurrency of 4.5% (Tables 8 and 9 in [32]). Those numbers may have understated concurrency by approximately 40% due to flawed questionnaire design (as described in [8]). In addition, point prevalence of concurrency is approximately half of one-year concurrency in 11 post 2009 DHS/AIS (from countries reported in Table 1). Adjusting the pre-2009 DHS data for those factors (increasing reported rates by 40% due to faulty questionnaire design and dividing by 2 to find point prevalence) produces an average point prevalence of 3.1%, close to the 3.4% found in post-2009 DHS/AIS shown in Table 1. (See Appendix I for calculations.) In summary, the data reported in Table 1 show that the UNAIDS protocol produces concurrency rates that are consistent with the results of other recent surveys in the region not using the protocol.

In the United States, reported men's one-year concurrency is 11–13% [10, 33] and women's is 5.2% [10]. The 11 DHS/AIS surveys in SSA listed in Table 1 report average men's and women's one-year concurrency (not shown in table) as 9.8% and 1.2%. The pre-2009 DHS one-year concurrency data (after adjusting for the methodological errors noted in the foregoing paragraph) show men's one-year concurrency as 11.4% and women's as 1.2%. Similar (but not strictly comparable) surveys from Europe [34] suggest that concurrency there is not very different from US levels. Furthermore, average point prevalence of concurrency in the 13 surveys reported in Table 1 is 3.4%, lower than the 3.6% in the United States [10]. Those data on concurrency are consistent with numerous other surveys finding comparatively conservative sexual behaviour in SSA (for example, [35]). The concurrency hypothesis – as formulated by Halperin and Epstein [11, 12] – asserts that concurrency is higher in SSA than elsewhere, but data from these surveys suggest otherwise.

### Measurement error

There is broad agreement that surveys understate the prevalence of concurrency and other sexual behaviours since some respondents are unable or unwilling to answer questions correctly. (For recent examples, see [8, 36, 37].) Some researchers try to use biomarkers as proof of sexual experience despite respondents’ denials [38–40], but most biomarkers cannot provide definitive evidence of misreporting. Laboratory tests for HIV or HSV-2, for example, do not have 100% sensitivity. Even if they did, the tests only determine if individuals are infected, not how they acquired the infection. (Both viruses can be transmitted non-sexually.) Even if biomarkers provide evidence of sexual contact, they do not show that the sexual exposure was with a concurrent partner. Researchers have also experimented with a variety of interview methods aimed at getting around respondent's reluctance to reveal stigmatized behaviour. One cannot assume, as some have [28, 41, 42], that the method yielding the higher reported prevalence of concurrency is the more accurate. Sexual behaviour is misreported in surveys, but biomarkers and improved questionnaires do not allow one to know with certainty the size and in some cases even the direction of reporting error.

The two most important sources of reporting error pertinent to the present discussion are imperfect memory and social desirability bias. Other errors (such as interviewers’ recording errors) are likely to be random and thus not affect comparisons of concurrency between populations. The objective of this section is to identify the likely important and systematic sources of under-reporting of concurrency in SSA and to search for clues about the possible size of the bias.

### Recall error

One form of recall error is “heaping” or an unusually high frequency of reported behaviours on the same date (for example, “six months ago”). That can produce over-estimation [43] or under-estimation of partnership overlap [44]. Heaping at six months prior to the interview could produce *over*-reporting of concurrency using the UNAIDS protocol, which measures point prevalence of concurrency at six months. Another source of recall error arises if recent memories of sexual activity are more accurate than distant ones. That can take the form of “telescoping” if respondents report distant events as more recent than they were.

Measuring concurrency at the time of the interview would appear to reduce recall error from heaping or telescoping. Nevertheless, it requires respondents to know whether there will be at least one more sexual encounter in reported overlapping partnerships, which, of course, the respondent cannot know. Some methods of measuring current concurrency also rely on respondents’ understanding of what constitutes an on-going sexual relationship, which is one reason why the UNAIDS panel of experts discourages measuring it. If wishful thinking about the future course of partnerships prevails over pessimism, current point prevalence will be over-reported.

In practice, heaping, telescoping and unwarranted optimism appear to produce reporting errors too small to affect our understanding of the role of concurrency in HIV epidemics. Eaton *et al*. [26] finds men's self-reported concurrency at the time of the interview to be 6.7%, just 2 percentage points higher than what respondents reported six months before the interview. Eaton *et al*. measure point prevalence of concurrency at monthly intervals during the year before the interview. If concurrency trailed off in successively more distant months, then recall error would be the likely explanation. Nevertheless, they find that point prevalence of concurrency varied in a narrow 0.5 percentage point range between two and eight months prior to the interview, bracketing six-month reference point used in the UNAIDS protocol. Glynn *et al*. [28] found men's current point prevalence to be 0.5 percentage points lower than point prevalence measured six months earlier. In addition, Brewer *et al*. analyse the results of five studies in which both partners reported dates of sexual exposures. They find that “the absence of telescoping and consistent heaping suggests reported dates of exposure are largely free of these 2 common types of response error” [45]. (See also [46].)

One form of recall error arises when respondents forget about partnerships. Short-term encounters, especially distant ones, appear to be the most easily forgotten [28, 44]. Helleringer *et al*. report that “long-term concurrent partnerships … may be less prevalent than initially thought. ‘Experimental’ or ‘transitional’ concurrent partnerships … may also represent common types of concurrency in sub-Saharan settings” ([44], page 519). Failure to report one-time or very short partnerships occurring more than six months before the interview does not affect six-month point prevalence of concurrency. Moreover, except during acute infection, per-act transmission rates of HIV are vanishingly small; thus long-term partnerships, not sporadic, one-time sexual encounters are likely to account for the preponderant share of incident infections [11, 12]. If long-term partnerships are key to understanding the importance of concurrency, then the failure to remember short-term partnering would appear to be of little import if the reason for measuring concurrency is finding support for the concurrency hypothesis.

In summary, the foregoing discussion indicates that recall error is unlikely to produce important under-reporting of concurrency in surveys using the UNAIDS protocol.

### Under-reporting due to social desirability bias

The most intractable reporting errors appear to come, not from respondents’ poor memory, but from their unwillingness to answer questions truthfully [39, 44, 47, 48]. Stigmatisation of concurrency can lead respondents to under-report concurrency. In circumstances where revealing concurrency can lead to shaming, shunning, divorce (and thus the loss of one's children or one's land) or even physical assault, it is likely that stigmatization reduces both the willingness to report concurrency and the inclination to engage in concurrency, leading to low levels of reported concurrency for both reasons. The multiple effects of stigmatization complicate the analysis of the under-reporting of concurrency. In contrast, positive attitudes about concurrency can lead to over-reporting, for example, when sexual exploits are admired or when sexual activity is considered a badge of honour or rite of passage to adulthood. In addition, if long-term relationships are less stigmatized than brief encounters or if high status partners are prized, some men and women respondents might exaggerate the number of their partners or the length of their partnerships, both of which produce over-reporting of concurrency.

Some studies provide hints about the possible size of net under-reporting of women's concurrency. Helleringer *et al*. [44] examined partnerships reported by either or both partners. Among women, 4.6% self-reported concurrency. If men correctly reported their partners, then 11.1% of women should have reported concurrency, that is, concurrency was under-reported by about 60%. Under-reporting by women would have been 30% if men over-reported by the same amount that women under-reported. Gregson *et al*. found that women aged 15–49 years marking their own questionnaires and putting them in a locked box were about five times more likely to report concurrency (adjusted odds ratio of 5.24) at the time of the interview than other respondents reported in face-to-face interviews ([49] page 572). If one assumed that the locked-box interview produced accurate reporting and that actual concurrency was the same in the two groups, then women could have under-reported concurrency by about 80% in the face-to-face interview. Mensch *et al*. report that unmarried girls aged 15–21 years were 2.35 times more likely to report ever having had concurrent partners in computer-assisted interviews than other respondents of the same age and gender reported in face-to-face interviews ([39] page 257). Together, these studies suggest that women's under-reporting due to social desirability bias could range as high as 30–80%.

While there appears to be considerable agreement that women understate their concurrency in surveys, it is less clear whether countervailing effects of men's “swaggering” or embarrassment lead to net over- or under-reporting of concurrency. Helleringer *et al*. [44] find that men could have net over-reported concurrency by as much as 240%, assuming women correctly reported their partners. Both Nnko *et al*. [47] and Morris [50] find that men report more partnerships than is possible if women do not under-report partnerships. Mensch *et al*. report that different interview methods produced no statistically significant difference in reported concurrency for boys aged 15–21 ([39] page 257). Gregson *et al*. report that men aged 15–49 years marking their own questionnaires and putting them in locked boxes were one third more likely to report concurrency (adjusted odds ratio of 1.33) at the time of the interview than other respondents of the same age and gender reported in face-to-face interviews ([49] page 572). If the locked-box method produced accurate results and the two groups of men had identical levels of concurrency, then men could have under-reported concurrency by about 25% in face-to-face interviews. In summary, evidence suggests that there could be substantial net over-reporting of concurrency by men or that they might net under-report concurrency by as much as 25%.

### Qualitative studies and under-reporting

Some argue that qualitative research using recruited respondents casts doubt on the results of survey research. Epstein and Morris assert, “qualitative studies of small population samples consistently find that respondents report engaging in concurrent partnerships at rates that are often many times higher than in behavioural surveys” [8]. None of the studies they cite, however, supports that assertion. One cited study reports point prevalence of concurrency of 4.2% among adults in Botswana [51]. Another study reports that about 6% of South African youth aged 20–30 had two or more, but not necessarily concurrent, partners in the previous month [52], suggesting that concurrency among those respondents was consistent with the data reported in Table 1. None of the other studies [53–57] reports *any* rates of concurrency and thus could not have reported rates of concurrency that are “many times higher than in behavioural surveys”. The authors of some of the studies cited by Epstein and Morris [52–54, 56] state that concurrency is “common”, but the word has no quantitative denotation or connotation that can be used in evaluating the results of quantitative research.

Qualitative research can be valuable in addressing some issues. As Hogle and Sweat put it, “qualitative methodologies attempt to answer the ‘why’ questions and deal with the emotional and contextual aspects of response, adding ‘feel,’ ‘texture,’ and nuance to quantitative findings” [58]. Another source says that qualitative evidence can show “how and why people behave, think, and make meaning” of their lives, and it falls “within the context of discovery rather than verification” [59]. What participants in qualitative research believe about the behaviour of other members of their community may help answer some research questions. There are valid reasons to suspect that representative surveys of defined populations systematically under-report concurrency. Nevertheless, qualitative research is unlikely to be helpful in determining the extent of under-reporting.

### How much does reporting error matter?

Asserting that actual concurrency is “many times higher” than reported concurrency is not useful for policy making unless one is able to guess about how many is “many”. Table 2 presents the results of making guesses about the extent of reporting error, guesses based on the discussion in foregoing paragraphs.

The objective of this exercise is to suggest the maximum plausible levels of concurrency in the 13 countries in Table 1 that report concurrency for both genders using the UNAIDS protocol. The 6 columns in Table 2 represent different assumptions about the under-reporting of concurrency due to social desirability bias. They assume women net under-report concurrency by 0%, 60%, 90% or 95% and that men net under-report concurrency by 0% or 25%. Column No. 6 makes no specific assumption about the proportion of concurrent partners women do not report due to social desirability bias, but instead assumes that women's concurrency is equal to two-thirds of men's. In countries in which women report relatively high levels of concurrency (Lesotho and Cameroon), the assumption that women report only 5% or 10% of their concurrent partners leads to improbable outcomes. In Lesotho, for example it would mean that women's actual concurrency was 23% or 46%—even higher with recall error—and would exceed men's concurrency by a substantial margin. To avoid implausible outcomes such as these, women's concurrency in each cell of Table 2 is capped at two-thirds of men's. In addition, the three panels of 13 rows each represent different assumptions about recall error. They assume under-reporting of concurrency due to imperfect recall of 0%, 7.5% and 15%. Extrapolations from the table can accommodate larger hypothetical reporting errors if those assumed here are deemed too small. The hypothetical levels of concurrency as shown in Table 2 exceed 13% in only 2 countries (13.6% in Burkina Faso and 17.4% in Cameroon).

**Table 2:** Concurrency in 13 countries using UNAIDS protocol with hypothetical reporting errors

	Assume no social desirability bias	Assume under-reporting due to social desirability bias				
		
Country or District	No. 1	No. 2 Men report 100%, women report 40% of concurrent partners	No. 3 Men report 100%, women report 10% of concurrent partners	No. 4 Men report 75%, women report 10% of concurrent partners	No. 5 Men report 75%, women report 5% of concurrent partners	No. 6 Men report 75% of concurrent partners, women report 2/3 of men's concurrency
	*Assume no recall error by men and women respondents*					
Burkina Faso	5.3	5.3	5.7	7.4	7.9	11.6
Burundi	0.8	0.8	0.8	1.0	1.0	1.7
Cameroon	7.6	9.0	11.1	14.8	14.8	14.8
Ethiopia	1.2	1.2	1.2	1.5	1.5	2.6
Lesotho	4.9	6.6	6.2	8.2	8.2	8.2
Malawi	2.0	2.0	2.4	3.0	3.5	4.2
Mozambique	4.8	5.4	8.4	9.8	9.8	9.8
Rwanda	0.8	0.9	1.3	1.5	1.7	1.7
Senegal	2.7	2.8	3.6	4.4	5.4	5.7
Uganda	5.1	5.4	6.9	8.5	10.5	10.8
Zimbabwe	2.0	2.3	3.4	4.0	4.2	4.2
Uganda rural	5.1	5.4	6.9	8.5	10.5	10.9
Kwa Zulu Natal	2.6	2.9	4.4	5.1	5.2	5.2
	*Assume respondents forget to report 7.5% of concurrent partners*					
Burkina Faso	5.7	5.8	6.2	8.0	8.6	12.5
Burundi	0.8	0.8	0.8	1.1	1.1	1.8
Caneroon	8.2	9.8	12.0	16.0	16.0	16.0
Ethiopia	1.2	1.2	1.2	1.7	1.7	2.8
Lesotho	5.2	7.1	6.7	8.9	8.9	8.9
Malawi	2.1	2.2	2.6	3.3	3.8	4.6
Mozambique	5.2	5.8	9.1	10.6	10.6	10.6
Rwanda	0.9	0.9	1.4	1.6	1.8	1.8
Senegal	2.9	3.0	3.8	4.8	5.8	6.1
Uganda	5.5	5.8	7.4	9.2	11.3	11.7
Zimbabwe	2.2	2.5	3.7	4.4	4.6	4.6
Uganda rural	5.5	5.8	7.5	9.2	11.4	11.8
Kwa Zulu Natal	2.8	3.1	4.7	5.5	5.6	5.6
	*Assume respondents forget to report 15% of concurrent partners*					
Burkina Faso	6.2	6.3	6.7	8.7	9.3	13.6
Burundi	0.9	0.9	0.9	1.2	1.2	2.0
Cameroon	8.9	10.6	13.0	17.4	17.4	17.4
Ethiopia	1.4	1.4	1.4	1.8	1.8	3.0
Lesotho	5.7	7.7	7.3	9.7	9.7	9.7
Malawi	2.3	2.4	2.8	3.6	4.2	5.0
Mozambique	5.6	6.4	9.9	11.5	11.5	11.5
Rwanda	0.9	1.0	1.5	1.8	2.0	2.0
Senegal	3.1	3.3	4.2	5.2	6.4	6.7
Uganda	5.9	6.3	8.1	10.0	12.3	12.7
Zimbabwe	2.4	2.7	4.0	4.7	5.0	5.0
Uganda rural	6.0	6.4	8.1	10.0	12.4	12.8
Kwa Zulu Natal	3.0	3.4	5.1	6.0	6.1	6.1

The foregoing discussion suggests an inverse relation between the under-reporting of women's concurrency and actual levels of concurrent partnering. Such a correlation is consistent with the notion that both higher levels of concurrent partnering and lower levels of under-reporting of concurrency are likely, other things equal, where concurrency is less highly stigmatized. Where stigma attached to concurrency is higher, the opposite would hold. If those conjectures are correct, then the more plausible hypothetical rates of concurrency in Lesotho and Cameroon may be found in the columns No. 2 or 3 in Table 2 while the more plausible hypothetical rates of concurrency in countries such as Burkina Faso or Burundi are more likely in columns No. 5 or 6.

The exercise in Table 2, which examines the effect of hypothetical levels of reporting errors on concurrency in SSA, suggests that the actual prevalence of concurrency in the region ranges between 2 and 14%. The next step is to determine what modelling can tell us about HIV epidemics when concurrency is at or below the estimated upper bound concurrency prevalence of 14%.

### Changes in concurrency

Part of the controversy over concurrency is not about its level now, but its level during early years of the epidemics in SSA when HIV prevalence grew rapidly in many SSA countries. Attention in this regard has focused on Zambia, Zimbabwe and Uganda. Sandøy *et al*.'s study suggests that reported concurrency fell by about 30% from 1998 to 2003 in Zambia (Table 1 in [31]) and there is evidence of declines in other risky sexual behaviours [60, 61]. Other studies find downward trends in a variety of reported risky sexual behaviours in Zimbabwe [62–65], but the only direct evidence of declines in concurrency comes from a study in one province of Zimbabwe (Manicaland) where respondents reported fewer current partners in 2001–2003 than in 1998–2000 (Table S5 in the supplement to [62]). Some argue that risky sexual behaviour has also declined in Uganda [66–68]. A prominent nationwide campaign encouraging people to be faithful and engage in “zero grazing” aimed to reduce concurrency in Uganda, but evidence of the campaign's success from representative surveys is lacking. Cleland *et al*. [69] say that even tentative conclusions about trends in sexual behaviour require at least three surveys using the same question, but even Sandøy *et al*.'s [31] Zambian study only approximates that criterion.

It is plausible that, as deaths from AIDS increased and the nature of HIV became more widely understood, people in SSA chose to have fewer concurrent partners or that HIV-prevention programs persuaded people to have fewer concurrent partners. There may have been unrelated, long-term declines in risky sexual behaviour. All of these factors, however, could have produced increased stigmatization of concurrency – leading to reductions in over-reporting and/or increases in under-reporting – rather than or in addition to producing changes in concurrent partnering. We have almost no credible evidence that reported concurrency has declined in SSA in recent decades. Even if we did, we have no way of knowing whether changes in reported concurrency represent different behaviours or different amounts of reporting error. Accordingly, the effort to show that a decline in concurrency could explain the apparent drop in HIV prevalence in some countries of SSA is an exercise that is unlikely to succeed.

## Modelling and the concurrency hypothesis

Supporters of the concurrency hypothesis argue that concurrency is more effective than sequential partnering in spreading HIV [11, 13]. If that were not so, it would be difficult to construct a plausible argument for why the concurrency hypothesis could be correct. Since mathematical models are the most important way to build a case for the special ability of concurrency to spread HIV, much of the controversy over the concurrency hypothesis has centred on modelling HIV epidemics. Modelling cannot provide evidence of concurrency's capacity to spread HIV. It can only show that a given set of assumptions about sexual behaviour, viral infectivity and other factors is consistent with certain HIV epidemic trajectories. Only an examination of a model's assumptions, therefore, can reveal whether its simulations have any relevance to actual HIV epidemics.

### More realistic transmission rates

Eaton, Hallett and Garnett [70] make a key contribution to the debate over the concurrency hypothesis since their model addresses an important drawback of Morris and Kretzschmar's model [71] (hereafter the M-K model), which played a pivotal role in launching the hypothesis in the 1990s. Eaton *et al*.'s most significant modification of the M-K model is replacing Morris and Kretzschmar's transmission rate, which has drawn especially critical commentary [1–4, 72]. Their transmission rate is apparently based on a study of soldiers and commercial sex workers in Thailand [73, 74]. Despite considerable criticism of their transmission rate, which is far higher than used by other modellers, Morris and Kretzschmar have never explained why their choice of transmission rate is appropriate.

Eaton *et al*.'s transmission rate is based on calculations by Hollingsworth *et al*. [75], who rework Wawer *et al*.'s data [76] from a study in Rakai in the 1990s. Their daily transmission rate is far lower than Morris and Kretzschmar's and varies according to stage of HIV infection. (The virus is more infective in the early months of the infection.) Morris and Kretzschmar simulated their model for only five years, but Eaton *et al*.'s transmission rate is so low that simulated HIV prevalence hardly changes in five years. To see how the model would perform over a longer period, they incorporated vital dynamics into the model by allowing for deaths from AIDS. They also accelerated their simulated epidemics by beginning with 1% HIV prevalence instead of Morris and Kretzschmar's 0.05% HIV seeding rate.

Morris and Kretzschmar report that when concurrency (the point prevalence of the average of men's and women's concurrency) is 12% (at which point half of partnerships are concurrent), their model produces a 900-fold increase in simulated HIV prevalence in five years, rising to 45%, which “is 10 times as large as under sequential monogamy” ([71] from the abstract). Eaton *et al*.'s parameterization produces dramatically lower epidemic trajectories. With concurrency at 12%, it takes almost 100 years for HIV prevalence to reach 5%. Moreover, “with staged transmission and up to 8% of individuals having concurrent partnerships, HIV fails to spread” [70], that is, simulated HIV epidemics are unsustainable and move to extinction. Unless concurrency exceeds 8%, curves that depict simulated HIV epidemics (figure 1b in [70]) lay almost on top of each other, that is, concurrency makes essentially no difference to HIV epidemic trajectories. Eaton *et al*. sum up the results of their modelling by saying, “this model produces HIV epidemics that grow more slowly than those observed in southern Africa” [70].

### Increasing the realism of Eaton *et al*.'s parameterization

Eaton *et al*. [70] find dramatically slower HIV epidemic spread than Morris and Kretzschmar, but even that slow growth overstates what a properly parameterized model generates.

#### Transmission rate that is unrealistically high

As noted, Eaton *et al*.'s transmission rate is based on data collected by Wawer *et al*. [76], who studied HIV transmission in stable discordant couples. Their analysis accounts for the presence of genital ulcer disease, but they do not consider the effects of coinfections other than STIs on transmission efficiency. There is substantial evidence that *Schistosomiasis hematobium*, malaria and possibly other coinfections raise HIV transmission rates [77–86]. If that were the case, coinfections would have produced an upward bias in Eaton *et al*.'s estimate of transmission risk. If so, their simulations represent the combined impact of concurrency and coinfections, not the result of concurrency *per se*, that is, their results overstate the importance of concurrency. Boily *et al*. make the same point more generally, concluding that “the role of concurrency in Africa may have been overestimated because of the high prevalence of HIV cofactors” [87].

#### Partnership duration

One of the parameter values in the M-K model [71] that Eaton *et al*. did not change was average partnership duration of 200 days. Evidence on partnership duration in SSA is not abundant. Much of it comes from studies of young people whose partnerships are necessarily short [27, 88, 89], studies of non-spousal partnerships [47, 90] or studies that recruited rather than sampled respondents [91]. A single representative survey that reports the duration of both primary and secondary partnerships for adults could be found [10]. In that survey in Rakai, Uganda in the early 1990s, the average duration was 20 years among married respondents and just over 17 years among all respondents ([73]; see also [8].) Short partnership duration produces high rates of partnership turnover, which spreads HIV rapidly [2]. In 2000, Morris and Kretzschmar [73] published a version of their 1997 model parameterized with data from the Rakai survey [10]. Their new assumption of longer average partnerships led to dramatically lower epidemic trajectories. With concurrency just over 12%, HIV prevalence rose from 1% to only 2.5% in five years, not from the 0.05% to 45% that they found in 1997 with 200-day partnerships (figure 3, scenario 9 in [73]). (In addition to longer partnerships, they also assumed gender asymmetry of concurrency, but as noted earlier, that does not have an important effect on simulated HIV epidemics.)

Lengthening the duration of partnerships has a similar effect in Eaton *et al*.'s model [70]. Eaton *et al*. originally found that, with the average partnership at 200 days and concurrency at 12%, simulated HIV prevalence reached a maximum of about 10% in 250 years. Alan Isaac and I simulated Eaton *et al*.'s model, increasing average partnership duration from 200 days to three years (less than a fifth as long as found in Rakia by Morris *et al*. [10]). With longer partnerships, HIV epidemics generated by the model were unsustainable (they moved to extinction) unless concurrency exceeded 12%. Increasing average partnership duration in Eaton *et al*.'s model to four years leads to epidemic extinction at any level of concurrency up to 15%; with average partnerships at five years, the model produces epidemic extinction at any level of concurrency up to 18%.

#### Coital dilution

My colleagues and I have argued elsewhere [5] that Eaton *et al*.'s parameterization exaggerates the importance of concurrency in a third way, by failing to incorporate coital dilution, which is the lower average coital frequencies in secondary partnerships. Both the M-K model [71] and Eaton *et al*. [70] assume that adding a second partner doubles one's coital frequency, a third partner triples one's sexual activity and so on. The empirical evidence for coital dilution is thin, but there appears to be no contrary evidence (see [92] for recent evidence and [5] for other citations).

Sawers, Isaac and Stillwaggon [5] simulate Eaton *et al*.'s model with the level of coital dilution that Morris *et al*. report in Rakai, Uganda [10]. Doing so generates HIV epidemics that move rapidly from the initial HIV prevalence towards zero prevalence at every level of concurrency considered, and move to extinction more rapidly at higher levels of concurrency. In other words, concurrency is protective against HIV at the population level. Sensitivity analysis shows that even with much lower levels of coital dilution than reported by Morris *et al*. [10]) in Rakia, HIV epidemics are not sustainable at any considered level of concurrency.

We next simulate Eaton et al.'s model incorporating both longer partnerships and coital dilution at the same time. We increase mean partnership duration (from 200 days to three years) and allow for coital dilution (25% lower coital frequency in secondary partnerships compared with primary partnerships). With those modifications, simulated HIV epidemics are unsustainable at any level of concurrency up to 18%. With concurrency at 19%, HIV prevalence rises from 1% to less than 1.2% in a decade and only to 1.5% in a century. If concurrency is 22%, HIV prevalence does not reach 3% in 50 years. These lower bound estimates of the ability of concurrency to generate an increase in HIV prevalence are almost surely too low, given the conservative assumptions about partnership duration and coital dilution on which they are based. (In Rakai, Morris et al. found average partnerships of more than 17 years and average coital dilution of more than 75% [10]).

Recall that in Table 2, the plausible upper bound estimate of concurrency is below 14%. In short, the level of concurrency needed to avoid epidemic extinction in sexual network models patterned on the M-K model is far above plausible estimates of concurrency prevailing in SSA. This modelling is not consistent with the assertion that concurrency is an important explanation for the high prevalence of HIV in the region or was the principal driver of the dramatic increases in HIV prevalence in the early stages of many epidemics in the region.

### Early model overstates impact of concurrency on HIV

Morris and Kretzschmar's articles presenting their model [71, 73, 93, 94] have been cited in more than 1000 publications (according to Google Scholar) and were – to the exclusion of all others – repeatedly cited by the most prominent proponents of the concurrency hypothesis [11, 12, 95, 96], who have been, in turn, cited in hundreds of publications. Thus, the 15-year-old M-K model still plays an outsized role in the debate over concurrency and the realism of its simulations continues to be an important and contested issue. Epstein and Morris assert that the early “proof-of-concept” model of Morris and Kretzschmar produces “an *underestimate*, not an overestimate, of the effect of concurrency” ([8] emphasis in the original). Goodreau *et al*. [89] make the same claim. Kretzschmar says the M-K model's “conclusions drawn about the impact of concurrency are strong and are convincing” [97]. What follows examines those assertions.

Eaton *et al*. [70] modified the M-K model by incorporating vital dynamics, changing the transmission rate and increasing the seeding prevalence. Alan Isaac and I reversed the last two changes, retaining vital dynamics in order to analyse simulated epidemics for more than five years. In just 11 years of simulations, the M-K model modified only to include vital dynamics generates 99% HIV prevalence at every level of concurrency including serial monogamy, a result that does not track the epidemic trajectory of any known human disease. The dramatic differences in simulated HIV prevalence generated by different levels of concurrency are an artefact of truncating the simulations at five years. Extending simulations for just six more years, which adding vital dynamics allows, leaves concurrency with no effect on simulated HIV prevalence.

Morris and Kretzschmar's choice of transmission rate has been criticized [1–4], but the problem with their model is more accurately described as the interaction between the unrealistically high transmission rate and rapid partner turnover. Their 5% daily transmission risk produces a 99.996% chance of transmission in initially serodiscordant partnerships that last 200 days, the average partnership duration in their model. In the M-K model, after partnerships dissolve, new ones quickly form (in 100 days on average). The virus is “trapped” in neither sequential nor concurrent partnerships due to the short duration of each partnership and the brief interlude between them.

Epstein and Morris predicted that allowing for deaths from AIDS (adding vital dynamics to the M-K model) would accelerate the simulated spread of HIV—which it does—and increase the impact of concurrency [8]. The reason they give is “because in the ‘serial monogamy’ scenario—but not in the concurrency scenario—most infected individuals die before they can infect at least one other person” [8]. That is incorrect. Given the assumptions of the M-K model, serially monogamous individuals do transmit the virus to many others before they die. In a scenario in which all partnerships are sequential and each lasts 200 days, an individual once infected will have 10 or more additional partners—likely infecting all of them—before dying from AIDS 9.4 years later. If one modifies the M-K model to account for vital dynamics, any death from AIDS considered in isolation reduces HIV prevalence since it reduces the number of individuals living with HIV. Nevertheless, the premature death frees the surviving partner—almost surely infected with HIV, given the model's assumptions about viral infectivity—to form new partnerships, which spreads the infection to others and accelerates the epidemic. Vital dynamics thus promotes the spread of HIV in the M-K model because the death of a partner increases the rate of partnership turnover.

To show the effect of Morris and Kretzschmar's transmission rate on their model's outcomes, we exchange their daily transmission rate for the one used by Eaton *et al*. [70]. Morris and Kretzschmar's 0.05 daily transmission risk is 89 times the size of Eaton *et al*.'s unstaged daily transmission risk of 0.00056 (which is the weighted average of transmission risks at different stages of the infection). The smaller transmission rate produces a 10.6% risk of transmission in 200 days, not the nearly 100% chance of transmission that Morris and Kretzschmar's transmission rates produces. Even with unrealistically rapid partner turnover, Eaton *et al*.'s lower daily transmission risk produces a dramatically slower growth path of HIV prevalence. In five years of simulations, HIV prevalence rises from the initial (seeding) 0.05% level to 0.06% at all levels of concurrency including serial monogamy. In 11 years, HIV prevalence grows to either 0.07% or 0.08% depending on the level of concurrency, not the 99% that Morris and Kretzschmar's transmission rate produces. When concurrency is 12%, it takes over a century for HIV prevalence to rise to 3% (not to 45% in five years). Substituting staged for unstaged transmission rates slows the growth of simulated epidemics still further. (Compare figure 1a and 1b in [70].)

The simulations presented in the foregoing paragraphs show that the criticisms of Morris and Kretzschmar's original parameterization [1–4] were correct. Simulating Morris and Kretzschmar's model using empirically supported transmission rates dramatically slows the rate of growth in HIV, reduces the maximum level of HIV that concurrency can generate and eliminates the impact of concurrency except when it is highly prevalent. Accounting for the effect of coinfections in raising transmission rates would further reduce the impact of concurrency in sexual network models. Incorporating coital dilution and longer partnerships in the M-K model undermines still further the ability of concurrency to drive the growth of simulated HIV epidemics. In short, the M-K model does not produce “an *underestimate* … of the effect of concurrency” [8], but instead greatly overstates its impact.

### Other recent sexual network models and the concurrency hypothesis

We have shown that, when properly parameterized, the M-K model [71] and its derivatives [5, 70] cannot generate sustainable HIV epidemics without assuming unrealistically high levels of concurrency. One must consider whether that result is produced by some particular characteristic of the M-K-type model not found in other models. For example, the M-K-type model is relatively simple compared with many recent models and more complicated modelling might generate simulated epidemics that avoid extinction. Appendix II contains a review of recent sexual network models that examine the effect of concurrency on HIV epidemics [89, 98–104]. The review finds that models cannot produce results consistent with the concurrency hypothesis without assuming unrealistic parameter values.

An example of a recent model that is inconsistent with the concurrency hypothesis is Goodreau *et al*.'s [89]. Their parameterization is based on a survey of 18–30-year olds in Zimbabwe [105], and thus their results cannot be generalized to the adult population of the country since, as Goodreau *et al*. admit, average partnerships are much shorter among youth than among all adults [89]. Moreover, among those aged 18–30, reported concurrency (7.3%) in the survey used by Goodreau *et al*. in which respondents were recruited is six times higher than reported in a nationally representative survey carried out by the DHS (page 195 in [24]). Simulated epidemics generated by Goodreau *et al*.'s model were very close to the persistence threshold, that is, concurrency could barely prevent simulated HIV epidemics from moving to extinction. With more realistic concurrency prevalence, their model would be even less likely to simulate sustainable HIV epidemics – even for those aged 18–30 – and thus is not consistent with the concurrency hypothesis.

### Comparing concurrency only with serial monogamy

Embedded in the discourse over concurrency during the last two decades and continuing in recent contributions to the debate is a default counterfactual – serial monogamy – to which concurrency is almost always explicitly or implicitly compared. (Exceptions [102, 103] model HIV-prevention programs that reduce but do not eliminate concurrency.) Of course, there are only two kinds of multiple partnering – with and without overlapping partnerships – so the dichotomization is analytically useful. Nevertheless, there are no countries where multiple partnering is exclusively sequential. Comparing concurrency with sequential partnering (instead of comparing one level of concurrency with a different level) leads to an exaggerated perception of concurrency's importance. Using sexual network models to compare a country like Lesotho (with 4.9% point prevalence of concurrency [29]) to the United States (with 3.6% point prevalence [10]) generates only a trivial difference in simulated HIV prevalence between the two countries, not the 40-fold difference in actual HIV prevalence. That is so even if under-reporting means that the 4.9% and 3.6% are substantial underestimates of true levels of concurrency.

## Measuring the correlation between concurrency and HIV

Conceptually, having a concurrent partner does not raise an individual's risk of acquiring HIV any more than having a non-concurrent partner [9, 10]. Not surprisingly, most research does not find a correlation between one's own concurrency and one's own risk of HIV infection. On the other hand, an individual whose partner has one or more other partners must be at increased risk of acquiring HIV if the partner's partner has any possibility of being or becoming infected.

Attempts to find a measurable individual HIV acquisition risk from one's partner's concurrency have been unsuccessful. Perhaps the most convincing study to find no correlation is Tanser *et al*. [106]. They measured the number of partners and concurrency of men who lived in the immediate neighbourhood of female respondents. They found no correlation between women's HIV incidence and the level of concurrency among men living in the vicinity, but did find a correlation with the number of partners of men in the neighbourhood. Women could have had partners from outside the neighbourhood, but the alternative research strategy (mapping sexual networks) is fraught with its own problems. Thus, the study is not definitive but is the most robust test yet. Maher *et al*. also find no correlation between men reporting concurrency (measured using the UNAIDS protocol [14]) and HIV prevalence among their wives [25]. (See also [107].) Steffenson *et al*. find no correlation between prevalent HIV infections and partner's concurrency among sexually active youth of both genders in South Africa [108]. It seems logical that one's partner's concurrency should raise one's risk of HIV infection, but the effect may simply be too small to measure.

If concurrency were to raise an individual's risk of acquiring HIV by a significant amount, an appropriate policy response might be to change the HIV-prevention message to encourage people to have fewer concurrent partners. Finding a simple and effective way to prioritize concurrency reduction in the prevention message has proved elusive. That is why some have argued that it is time to put the concurrency debate to rest [109].

### Population risk

Establishing whether or not concurrency raises individual risk of HIV acquisition is important for another reason. The concurrency hypothesis is an assertion about population risk, not about individual risk. Nevertheless, for concurrency to produce an increase in HIV incidence at the population level, it must also raise risk at the individual level. In other words, for the concurrency hypothesis to be correct, it is a necessary condition that concurrency increase individual risk, and it must increase it by enough to explain HIV prevalence in parts of SSA that is 100 or 200 times the level found in most of the rest of the world. (Note that higher individual risk is not a sufficient condition for the concurrency hypothesis to be correct: concurrency can increase individual risk but have no effect or even be protective against HIV at the population level.) The failure to find a measurable HIV acquisition risk imposed by concurrency at the individual level thus undermines the plausibility of the concurrency hypothesis.

In 1995, the Global Program on AIDS (GPA) of the World Health Organization released the results of sexual-behaviour surveys in four countries (and one city) in SSA [110]. Those were the first nationally representative surveys using a consistent definition of concurrency across so many countries in the region. Men's reported concurrency ranged from 13% in Kenya to 55% in Lesotho. Women's reported concurrency (measured in only two countries) was 9% in Tanzania and 39% in Lesotho. Concurrency in two countries in three cities in Asia and South America was far lower. Finding such extraordinarily high levels of concurrency in SSA provided a powerful impetus to the concurrency hypothesis. For example, the GPA data were the only concurrency rates in SSA cited by Halperin and Epstein in their important article in *The Lancet* in 2004, which gave crucial momentum to the hypothesis [11]. The GPA surveys asked respondents if they had more than one regular partner at the time of the interview. The proportion of women reporting regular partners who also reported no sex with a regular partner in the previous year was as high as 24% [110]. Since one cannot acquire HIV from a “regular partner” with whom one does not have sexual contact, the UNAIDS panel of experts [14] designed a definition of concurrency that allows researchers and not respondents to determine what is meant by the term.

In 2001, the DHS began releasing the results of surveys in SSA that measured concurrency by asking respondents the dates of sexual contacts rather than whether they had regular partners, and a very different picture of concurrency in the region began to emerge. The DHS concurrency data, even before the UNAIDS protocol was devised in 2009 [14], provided no evidence of a correlation between concurrency and HIV at the population level, either within SSA or globally [32]. Also, other surveys do not show that concurrency is especially prevalent in SSA [4]. As noted earlier, levels of concurrency reported in nationally representative surveys since 2001 are about the same or lower in SSA than in Europe and the United States where HIV incidence is a fraction of its level in SSA. Within SSA, rates of concurrency and HIV prevalence show no correlation [32, 111, 112]. The country with the highest reported concurrency in SSA using the UNAIDS protocol (7.6%) is Cameroon and its adult HIV prevalence is 5.3% compared with 5.0% in SSA as a whole [113]. HIV prevalence in countries in which only 2% of adults report concurrent partners (Malawi and Zimbabwe) is 11% and 14.3%, respectively, which is among the highest in the region. An ordinary least-squares regression of HIV prevalence on concurrency for the 11 countries in Table 1 does not find a statistically significant correlation between the two variables. (The *t* statistic on the regression coefficient is 0.70, far below statistical significance.)

Finding no population-level correlation between concurrency and HIV should not come as a surprise. With even small levels of coital dilution (lower coital frequency with secondary partners than with primary partners), one should expect an inverse relation between concurrency and HIV at the population level. If the number of partnerships in a population is fixed and the amount of concurrency increases, existing partnerships must be redistributed within the population. For every individual who acquires a partner, someone else must lose a partner. While some individuals form additional partnerships, an increasing share of the population is left with no partner. If coition is less frequent in second or third partnerships, concurrency by itself must reduce the frequency of sexual exposures, inhibiting the spread of HIV. That is so even if concurrency raises the risk of HIV acquisition at the individual level. Both empirical analysis and mathematical modelling support that reasoning [5, 92].

In summary, researchers have been unable to establish empirical evidence for a link between concurrency and HIV at either the individual or population level. Statistical analysis can only show the likelihood that a correlation exists, but cannot prove that it does not exist. Errors in measuring either HIV or concurrency would, if there is a correlation, bias downward its statistical significance and make it more difficult to observe. Confounding could also obscure the relationship. Boily *et al*. [87] discuss other methodological challenges to finding the HIV-concurrency correlation. Thus, the failure to find the elusive correlation cannot by itself end the controversy over concurrency.

### Outside-infection share

Several recent works offer what their authors present as a new way to link HIV and concurrency [8, 10, 114–116]. Epstein and Morris claim that “whether new infections arise from inside or outside the couple” can show whether “concurrency is a key driver of HIV epidemics in generalized epidemics in Africa” [8]. They take the proportion of incident infections in stable couples that come from outside the partnership in sub-Saharan Africa (which they calculate to be 60–84%) as confirmation of the importance of concurrency and “direct empirical evidence” for the concurrency hypothesis [116].

Epstein and Morris give no explanation for how the outside-infection share can be evidence supporting the concurrency hypothesis. A recent study in India [117] shows the implausibility of their assertion. The study argues that the driving force for the HIV epidemic there are men who bring infection into stable couples via concurrency. Nevertheless, HIV prevalence in India (0.3% of adults in 2009) is far lower than in sub-Saharan Africa [113] even though the outside-infection share is roughly similar in the two regions. Furthermore, the outside infection share is dependent on other factors besides the level of concurrency, specifically the transmission rate within stable discordant couples and the share of infections that do not come from sexual exposure. (See Appendix III for an explanation.)

In summary, the concurrency hypothesis is a claim that HIV incidence and concurrency are correlated at the population level. After years of trying, no one has been able to provide empirical evidence of that correlation without relying on the GPA surveys from 1989 and 1990, whose measurement methods have been questioned. Substituting the outside-infection share for the missing correlation does not provide evidence for the concurrency hypothesis.

## Summary and conclusions

This article examines recently published evidence relevant to the controversy over the concurrency hypothesis. The data put forward in support of the hypothesis have used a wide variety of definitions of concurrency producing incomparable measures of concurrency. Critics of the hypothesis [4] argued that the data used by supporters of the hypothesis were invalid, and supporters of the hypothesis responded by challenging the data used by the critics [8, 43, 118]. Under the auspices of UNAIDS, efforts to settle on a single definition of concurrency and avoid some obvious pitfalls in measuring it came to fruition in 2009. Since then, there have been at least 15 surveys using the UNAIDS protocol, which moves us towards a resolution of the debate. The new protocol collects data that permit a variety of different measures of concurrency, not just the recommended one. That has produced an unprecedented ability to diagnose virtues and drawbacks of different ways of measuring concurrency and to understand better the extent and nature of measurement error.

Surveys using the UNAIDS protocol find point prevalence of concurrency for adults aged 15–49 ranging between 0.8% and 7.6% in national and subnational surveys in SSA. Adjusting those data for plausible levels of reporting error produces an estimated range of 2%–14%. At issue is whether models assuming concurrency at that level (or even much higher) can generate sustainable HIV epidemics. The discussion begins with Morris and Kretzschmar's 1997 [71] (the M-K model) path-breaking and extraordinarily influential model and then looks at the effect of improvements in their model by Eaton *et al*. [70], Sawers *et al*. [5], and the results of modelling appearing first in this article. The M-K model reparameterized with conservative estimates of transmission rates, partnership duration and coital dilution generates sustainable HIV epidemics only when concurrency exceeds 18%, which is far above plausible levels. With concurrency at 21%, it takes 100 years of simulations with the modified M-K model for HIV prevalence to increase from 1% to 2.5%. With more realistic assumptions about transmission rates, partnership duration and coital dilution, concurrency would have to be even more prevalent to simulate sustainable HIV epidemics. Thus, the reparameterized M-K model generates simulated HIV epidemics inconsistent with the concurrency hypothesis at plausible levels of concurrency. A review of eight other recent mathematical models finds none consistent with the concurrency hypothesis (Appendix II).

Instead of looking at levels of concurrency and models of HIV epidemics to determine the validity of the concurrency hypothesis, some have tried to build the case by looking directly for the correlation between HIV incidence and concurrency posited by the concurrency hypothesis, but efforts to find a statistically significant correlation at both the individual and population level have so far been fruitless.

The notion that concurrency might play a special role in promoting the spread of HIV was first proposed in the early 1990s [119, 120], but Halperin and Epstein's 2004 article in *The Lancet* [11] gave new prominence to the hypothesis just as evidence was accumulating that the prevalences of other potentially risky sexual behaviours in the region were not exceptionally high [35]. On the basis of surveys in 1989 and 1990 from the GPA, they argued that concurrency was far higher in SSA than elsewhere. On the basis of simulations of the M-K model, they argued that HIV prevalence grows exponentially when concurrent partnering is common, but not when all partnerships are sequential. They coined a powerful metaphor to explain their argument, saying that serial monogamy “traps the HIV virus within a single relationship” [11], whereas concurrency allows it to spread quickly. This article shows that none of those assertions is correct. Concurrency is not especially high in SSA. When realistically reparameterized, the M-K model generates unsustainable simulated HIV epidemics at levels of concurrency that are empirically defensible. Finally, the virus is not “trapped” in sequential partnerships in the M-K model because of its assumed high transmission rate and rapid partner turnover.

One cannot prove that the concurrency hypothesis is incorrect, but the dearth of evidence in its support suggests that other explanations for SSA's extraordinary HIV epidemics should be considered.

### Beyond concurrency

Alternatives to concurrency as an explanation for SSA's HIV epidemics are at hand. One such possibility builds on scores of scientific studies that point to the role of coinfections that increase the efficiency of HIV transmission in sexual and vertical exposures, most prominently schistosomiasis [77–82], malaria [83–85] and STIs in promoting HIV transmission. (For a recent survey of the evidence, see [86].) Boily *et al*. [87] argue that confounding by coinfections could have exaggerated concurrency's importance in both empirical studies and in modelling exercises. Putting the same point differently, coinfections compete with concurrency as an explanation for sub-Saharan Africa's extraordinarily high HIV prevalence [121].

Mathematical modelling shows that even small increases in transmission efficiency could produce a substantial upward shift in simulated HIV epidemic trajectories. Eaton *et al*. (figure 1b in [70]) portray epidemics that reach a maximum HIV prevalence of 0–16% at different levels of concurrency. In the supplement to their article, however, they show that raising transmission rates by only 46% increases maximum HIV prevalence to 13–34%. In multi-burdened populations where people have chronic schistosomiasis and untreated chlamydia plus frequent bouts of malaria, transmission rates could easily rise by far more than 46% since the effects of different coinfections on transmission are likely to be additive or multiplicative. Models that have explicitly allowed for higher HIV transmission rates in sexual exposures due to coinfections find simulated epidemics with higher and more rapidly growing HIV prevalence [104, 122–127]. Modellers of HIV epidemic dynamics would do well to follow Boily *et al*.'s [87] advice and use their models, as others have, to examine the effect of risk factors that are not sexual behaviours but impinge upon sexual transmission.

In contrast, some authors have tried to discourage inquiry into the role of coinfections in HIV epidemics in SSA [8], saying “over the three decades since the AIDS pandemic first emerged, the field has been plagued by highly publicized ‘controversies’ driven by ideological advocates, some of whom have proposed that non-sexual drivers associated with poverty explain the extreme disparities in HIV prevalence within and between countries”. Readers should find the passage disturbing, in part because its authors erroneously identify coinfections that promote HIV transmission in sexual exposures as “non-sexual drivers of the epidemic”. Far more importantly, it suggests that research scientists, epidemiologists, clinicians and social scientists studying how diseases especially prevalent in low-income countries interact with transmission, progression and treatment of HIV are “ideological advocates” whose work is “a dangerous distraction”. The attempt to discourage inquiry that lies outside the narrow field of sexual behaviour by labelling it ideological and dangerous is an obstacle to finding the answers needed.

### HIV-prevention programming

In the effort to slow the spread of HIV in SSA, pivoting from an emphasis on sexual behaviour in general and concurrency specifically could lead to important changes in HIV-prevention programming and HIV-treatment protocols. For example, public health campaigns to reduce schistosomiasis, malaria, and STIs would be considered as HIV-prevention measures if coinfections were seen as important drivers of the epidemics. HIV-treatment protocols would include treatment and prevention of coinfections to reduce the contagiousness of those who are infected.

The extended debate over whether or not to include concurrency in HIV-prevention messages misses the far more important point that prevention policy is already too narrowly focused on sexual behaviour. Even if the concurrency hypothesis were correct, risky sexual behaviour is only one dimension of personal risk, only a single aspect of peoples’ very complicated lives [128]. Messages about sexual behaviour change are compatible with and are reinforced by messages about other health-promoting behaviours. Indeed, they could be the best way to get people to practice safe sex because those messages address the whole person instead of a single isolated aspect of their lives. People need information about treating and preventing coinfections that arguably promote HIV transmission and they need information and encouragement to demand safe and effective medical care and to know how to avoid other blood exposures that could transmit the infection. Such a message makes safe sex part of a broad health promotion program that encourages personal agency, empowering people to take charge of protecting themselves and their loved ones.

## Appendix I

Epstein and Morris argue that pre-2009 DHS used flawed methodology that understates concurrency, but the “problems appear to have been fixed in [the] 2009 … DHS from Lesotho” (page 11 in [8]). In addition to Lesotho, five other countries in SSA have DHS with one-year concurrency measured both before 2009 using the old questionnaire [32] and after 2009 (see Table 1) using the UNAIDS protocol. One-year concurrency in those six surveys (four for women) averaged about 40% higher than reported in pre-2009 DHS in the same countries, corroborating Epstein and Morris's assertion that the DHS concurrency measures of concurrency were too low. If all eight pre-2009 DHS in SSA that measured one-year concurrency [32] understated it by that same amount, then one-year concurrency for men and women in the eight surveys would have averaged 6.3% (that is, 40% higher than the 4.5% that was reported).

The UNAIDS protocol allows calculation of both point prevalence and one-year concurrency. In the 13 DHS/AIS that report concurrency for both genders and use the new protocol, point prevalence of concurrency averages about half of one-year prevalence. That suggests that point prevalence in the eight pre-2009 DHS [32], had it been measured, would be about half of the 6.3% estimated in the previous paragraph or 3.1%. That is close to the 3.4% average point prevalence that recent DHS/AIS using the UNAIDS protocol find in SSA (Table 1).

These (admittedly rough) adjustments (adding 40% and dividing by 2) to the pre-2009 one-year concurrency measures suggest that the UNAIDS protocol generates measures of concurrency that are not very different from what earlier surveys have found.

## Appendix II

The discussion of sexual network modelling in the main text emphasizes the sensitivity of the simulations to the model's structure and parameterization. What follows examines those features in eight recent models to determine whether they provide support for the concurrency hypothesis.

*Leclerc et al. (2009)* say that “all parameters were derived from empirical population-based data. Results show that basic parameters could not explain the dynamics of the HIV epidemic in Zambia” ([98] from the abstract). Only by assuming empirically unsupported transmission rates and prevalence of commercial sex work could the modellers track the actual epidemic in Zambia. Leclerc *et al*.'s simulations assumed that 37% of men with regular partners had either or both a regular or casual concurrent partner, but their modelling does not appear to include concurrency by men who only had casual partners other than CSWs, a serious omission. The authors used the Zambian 2001 DHS [129] to parameterize their model. Nevertheless, they assume that 18% of men with a regular partner had more than one although the Zambian DHS reports the figure as 9.1% (Table 6.2 in [129]), which is only 5.3% of all men. Furthermore, they specify point prevalence of casual concurrency for men with regular partners using one-year concurrency prevalence reported in the Zambian DHS (Table 18.13 in [129]). Point prevalence is always smaller, often much smaller than one-year prevalence. As a result, the point prevalence of concurrency for men with regular partners based on the 2001 Zambian DHS is probably about half of the 37% that Leclerc *et al*. assumed. Even that is at odds with another survey in Zambia that used a methodology similar to the UNAIDS approach and found concurrency for all Zambian men in 2003 to be 7.4% [31]. Thus, the level of concurrency assumed by Leclerc *et al*. was likely 3 to 5 times the correct figure. Even with that exaggerated level of concurrency, the model still could not track the explosive growth in HIV prevalence in the early years of the epidemic in Zambia.

*Morris et al. (2009)* [99] try to use concurrency to explain racial differences in HIV prevalence in the United States. They find that, compared with serial monogamy, concurrency produces a larger “epidemic potential”, that is, a larger number of individuals who are potentially at risk of HIV assuming that transmission occurs in every discordant partnership. That is essentially the same transmission risk as in Morris's model with Kretzschmar [71] in which transmission occurred in 99.996% of partnerships of average duration. The authors consider such individuals to be in the “reachable path” of the initial infection. Real-world HIV transmission risks in the absence of coinfections, however, are so low that almost all chains of transmission quickly break off. Thus, Morris *et al*.'s “reachable path” is a misnomer since it does not refer to an outcome that is possible to reach in an actual HIV epidemic in the United States or anywhere.

*Johnson et al. (2009)* [100] build a model parameterized with data from South Africa. They divide the population into two groups based on different propensities for high-risk sex, which they define as commercial sex and concurrency. One source of data they use to specify the size of the high-risk group is Shisana *et al*. [130], from which they determine concurrency rates of unmarried men and women. Those data, however, do not appear in the published report, so could not be verified. The study (Table 3.25) does give point prevalence of concurrency for those aged 15 to 24 for both married and unmarried respondents (39.2% for men and 23.1% for women). One-year concurrency, which was not reported, would have to be much higher than the point prevalence that was reported and those who had any partner in the previous year would have to be much higher still. In the table (3.24), however, the study reports multiple partners in the previous year (27.2% for men and 6.0% for women aged 15 to 24). Prevalence of concurrency cannot be larger than prevalence of multiple partnering (which includes non-overlapping partnerships), so the report on which Johnson *et al*. depend is internally inconsistent. At any rate, data for those aged 15–24 do not represent all adults. Furthermore, Johnson *et al*.'s concurrency rates are far larger than those found in recent surveys in South Africa and other SSA countries, as reported in Table 1.

Johnson *et al*. also assume, on the basis of two articles published in 1990 (when HIV transmission was poorly understood), that per-act transmission rates are five or six times higher (depending on the index partner's gender) in non-spousal partnerships than in spousal partnerships. A search turned up no publications since 1990 other than Johnson *et al*. that asserts a causal relationship between per-act transmission rates and number of sex acts. (See [5] Additional File 1 for an extended discussion of this issue.) Given their model's extraordinarily high rates of concurrency and transmission, it is not surprising that they conclude that most HIV transmission in South Africa occurs in non-spousal partnerships and that concurrency accounts for “roughly three quarters of new HIV infections” in South Africa ([100] page 317).

*Goodreau, Morris and colleagues (2010)* [89] developed a model that uses parameter values calibrated with data from a Zimbabwean study that recruited [105] rather than sampled participants who were 18–30 years old and thus the study was neither representative nor a study of all adults. Less than a quarter of partnerships in the survey were cohabiting – not surprising given the youth of respondents, and those partnerships lasted only 16.4 months on average, also reflecting, as the authors acknowledge, the youthfulness of the surveyed population. Goodreau *et al*.'s parameterisation generated simulated HIV epidemics with substantially higher trajectories than they would have found if they had used parameter values based on a population that included those older than 30, whose partnerships last longer. More importantly, Goodreau *et al*. say that the point prevalence of concurrency among the respondents recruited for the survey used to calibrate their model was 7.3%. Nevertheless, a survey using a nationally representative sample in Zimbabwe found concurrency to be 1.2% for those ages 15–29 and 2.0% for those ages 15–49 [24], not 7.3%. Also, a 2008 national survey in Zimbabwe reports that only 6.4% of adults had 2 or more sexual partners (though not necessarily overlapping) in the previous month [131].

Despite their high concurrency rate and short partnership durations, Goodreau *et al*. find that “the epidemic in Zimbabwe is very close to the persistence threshold – small changes in either behaviour [concurrency] or infectivity [transmission risk] may be enough to push it into eventual extinction” [89]. Although finding the HIV epidemic in Zimbabwe teetering on the brink of extinction, Goodreau *et al*. say “as one moves from the early ‘proof of concept’ models to models that are more realistically parameterized, the effects of concurrency become larger, not smaller” (page 313). Goodreau *et al*.'s findings and the analysis presented in the main body of the article do not support that assertion. Morris and Kretzschmar's proof of concept to which Goodreau *et al*. refer generated HIV epidemics growing explosively, not, as in Goodreau *et al*., verging on extinction.

*Delva (2010)* [101] argues that it is difficult to track the rapid spread of HIV in South Africa in the 1990s with a model that includes only serial monogamy, so concurrency must explain the country's HIV epidemic. Since other factors could have explained the rapid growth, his modelling strategy cannot provide evidence about concurrency's role in South Africa. Delva assumes HIV can only be transmitted sexually, ruling out hospital-acquired infections or other non-sexual transmission. That could be an appropriate assumption if Delva's objective were to explore only the sexual spread of HIV, not an actual epidemic, which doubtlessly had a non-trivial amount of non-sexual transmission [132, 133]. Moreover, as in most models of HIV and sexual network dynamics, Delva seeds his model only once (page 107 in [101]). He does so despite considerable evidence that the epidemic entered South Africa in multiple ways on multiple occasions, as infected contract workers, immigrants, returning travellers or emigrants, traders, tourists and other visitors crossed the country's borders [134–142]. (See [143] for additional citations.) Delva's simulations thus fail to capture an important characteristic of South Africa's epidemic, its multiple seedings.

*Enns et al*. [102] use a stochastic network model of sexual behaviour and HIV to examine cost effectiveness of behaviour-change programs aimed at reducing concurrency in Swaziland, Tanzania, Uganda and Zambia. The authors do not measure concurrency, but “used the number of sexual partners reported in the past 12 months as a surrogate measure for the number of concurrent partnerships” ([102] page 3). One-year multiple partnering must be higher than one-year concurrency, which in turn is often double point prevalence of concurrency. Not surprisingly, assumed initial levels of concurrency in the four countries (8%, 16%, 11% and 11%) are far higher than found in surveys reported in Table 1. Citing Wawer *et al*. [76], the modellers assume without explanation different monthly transmission probabilities in different countries. If the biology of HIV transmission is the same in all countries, only coital frequency could affect the monthly transmission probability, but the only source the authors give for data on coital frequency is Wawer *et al*.'s study in Uganda [76], which has no data on Swaziland, Tanzania and Zambia. The monthly transmission rates are inversely correlated (R^2^=.94) with the assumed initial levels of concurrency. All but one of the eight monthly transmission probabilities (acute and chronic phases in four countries) are substantially higher than those used by Eaton *et al*. [70], who cite Hollingsworth *et al*.'s [75] reworking of Wawer *et al*. [76]. Moreover, the model uses the same monthly transmission risk in both spousal and non-spousal partnerships and thus does not account for coital dilution. The high transmission risks assumed by the model, especially in non-spousal partnerships, exaggerate the effect of concurrency reduction on HIV incidence. The authors conclude that reducing concurrency, especially in high-risk populations, could reduce HIV incidence. If that were correct, their results would offer indirect support for the concurrency hypothesis. The model's parameterization, however, is sufficiently problematic to cast doubt on their conclusions.

*McCreesh et al*. [103] look at the effect of reducing concurrency on HIV outcomes in a rural district in Uganda, where point prevalence of concurrency was reported to be 4.9%, which is consistent with the data presented in Table 1. Nevertheless, the model does not take into account coital dilution and thus, as the authors acknowledge, overstates the effect of concurrency reduction. The article does not report essential parameter values, such as HIV transmission rates or the difference between high and low levels of sexual activity, making it difficult to evaluate the model's results.

*Orroth et al*. [104] use the STDSIM model to re-examine the results of the four-city study [111], which found no correlation between HIV prevalence and concurrency. They adjust parameter levels in the model to explore the possibility that multiple partnering and concurrency in the cities with high HIV prevalence was much higher than reported. The model when adjusted for more prevalent sexual risk behaviour could not account for the variation in HIV prevalence among the four cities. They then modify transmission rates to account for observed levels of male circumcision and STI prevalence. With biological cofactors added, the model tracked closely the observed differences in HIV prevalence among the cities. This modelling supports the hypothesis that biological factors, not behaviour (including concurrency), explain SSA's HIV epidemics.

More sophisticated models than the ones discussed above may ultimately show that concurrency is a driver of SSA's HIV epidemics. Numerous authors have argued that transactional sex is unusually prevalent in SSA, affecting patterns of partnering and thus HIV transmission. (See [13] for example.) Others make a related argument, contending that patronage of CSWs is far more common in SSA than elsewhere and is key to understanding HIV epidemics in the region [144]. Leclerc *et al*. included patronage of CSWs in their modelling of concurrency, but as noted earlier, generate only unsustainable simulated HIV epidemics when CSW patronage is at levels reported in the modelled population [98]. Transactional sex has been studied almost exclusively in SSA so there is as yet no evidence that it is more common in SSA than elsewhere. Even if it were, no one has yet shown that it makes any difference to HIV epidemic trajectories anywhere. Furthermore, the best evidence is that patronage of CSWs in SSA compared with other countries is not exceptionally high [145]. So far, efforts to show that the commercialization of sex in SSA explains the region's extraordinarily high HIV prevalence have been unconvincing.

Some recent contributions to the literature on concurrency have discussed the importance of different kinds of concurrency on epidemic dynamics, including transitional, compensatory, reactive, experimental and long-term concurrency [44, 146, 147]. Kretzschmar and Caraël [97] argue that various patterns of concurrent partnering might affect HIV epidemics, but they identify only a single way in which concurrency in SSA differs from concurrency elsewhere: the high prevalence of formal polygyny, which they argue is *protective* against HIV transmission. The only modelling they present to show that patterns of concurrency can affect epidemic trajectories is Kretzschmar's work with Xiridou *et al*. [148, 149], who modelled a population of men who have sex with men (MSM). Nevertheless, the concurrency hypothesis is about heterosexual populations in which partnership turnover is strikingly lower and partnership duration far longer than among the MSM they modelled. Initial efforts to model heterogeneity in sexual behaviour in the study of chlamydia transmission appear promising [150]. Nevertheless, the assertion that heterogeneity in concurrency affects the contours of heterosexual HIV epidemics (excepting the inclusion of CSWs) is as yet only a matter of speculation.

Concurrency varies widely in SSA, not just from one country to the next, but from one city or district to the next. Even within small geographical units, sexual networks are segmented by age, race, ethnicity, sexual activity levels and other factors. Some have suggested that national rates of concurrency may obscure detail that could explain explosive growth of HIV in eastern and southern Africa. It is argued that high rates of concurrency could produce localized hotspots of HIV from which the infection is spread to the rest of the country's population even though national concurrency rates are low. For example, see Eaton *et al*. [70]. Their modelling could not generate simulated HIV epidemics that track actual epidemics in SSA, but they conclude that “small groups with greater number of sexual partners … [could accelerate] the spread of HIV”.

The reason why this ‘Trojan Horse Effect’ is unlikely to rescue the concurrency hypothesis is that HIV epidemics, initially concentrated in isolated geographic or behavioural groups, suddenly developed into generalized epidemics with 10% or 20% of adults infected only in SSA and nowhere else [151]. Models already tell us that high rates of partnership turnover and high rates of coition can accelerate the growth of HIV. Without assuming that transmission rates are unusually high – for example, due to coinfections – we do not have models that show how initially high HIV prevalence in geographically or behaviourally restricted sexual networks can spread rapidly through the rest of a population not characterized by rapid partner turnover and frequent sexual exposures. Even if we did, “small groups with greater number of sexual partners” are found in many places besides SSA, so showing that the Trojan Horse Effect only functions in SSA will be a challenge.

## Appendix III

The following explains why the outside-infection share does not provide evidence about the link between concurrency and HIV incidence. There are three sources of incident infection among stable couples. Let,

α=incident HIV infections from outside the couple among stable discordant couples

β=incident HIV infections transmitted between partners in stable discordant couples

π=incident HIV infections in concordant negative couples, all of which must come from outside the couple

Two outside-infection shares are defined as,

$$\text{outside}-\text{infection share among stable discordant couples}=\frac{\alpha }{\alpha +\beta }$$

$$\text{outside}-\text{infection share among all stable couples}=\frac{\alpha +\pi }{\alpha +\beta +\pi }$$

Note that all infections from outside the stable couple (α+π) come from concurrent sexual partners only if one assumes that the single possible route of HIV transmission is sexual exposure.

Suppose the transmission rate between partners in stable discordant couples falls, for example, from couples counselling that leads to increased condom usage. That would reduce the number of incident infections between discordant partners (β) even if there is no change in outside incident infections in discordant couples (α), that is, no change in concurrent partnering. Ignoring nonsexual transmission, the outside-infection share among discordant couples [α/(α+β)] must consequently rise since the fraction's denominator (α+β) falls with no change in α. Thus, the outside-infection share among all discordant stable couples can move independently of any change in concurrency or other sources of outside infections.

Decreasing incident infections within discordant partnerships (β) would also increase the outside-infection share for *all* stable couples [(α+π)/(α+β+π)] for the same reason. The numerator is unchanged, but as β and thus the fraction's denominator falls, the value of the fraction rises.

Celum *et al*.'s study of 3480 discordant stable couples [152] can help provide a sense of the numerical impact of the intra-couple transmission rate on the outside-infection share. The study team counselled couples on how to prevent HIV transmission, supplied them with condoms, sent them to clinics to receive anti-retroviral therapy (ART), which inhibits HIV transmission [153], and treated them for STIs that could promote HIV transmission. Genetic sequencing was used to show that over the two-year study, 38 incident infections among couples in the study came from the outside and 91 came from within the couple. Without efforts to reduce intra-couple transmission, transmission between partners would have occurred in an estimated 664 couples, not 91. (That estimate of 644 intra-couple incident infections assumes no index partner was still in acute infection at baseline, 24 months to follow-up, and a daily transmission risk in discordant stable couples reporting no outside partners from Hollingsworth *et al*. [75], using data [76] that predate ART.) In that case, the outside-infection share among those discordant couples would have been 5.4% (38 out of 664+38) without efforts to reduce transmission, not 29% (38 out of 91+38). These calculations show that, even if concurrency is unchanged, variations in the within-discordant-couple transmission rate can be substantial, producing large changes in the outside-infection share.

The foregoing means that Epstein and Morris have over-estimated their outside-infection share among all stable couples in SSA (60–84%) since their calculations assume Celum *et al*.'s 29% outside-infection share among discordant couples. Many of those in discordant stable partnerships in SSA as a whole do not receive couples counselling, regular doctors’ visits to diagnose and treat STIs or ART. Thus, the transmission rate within discordant couples in the region is almost surely higher than among participants in Celum *et al*.'s study and thus the outside-infection share among discordant couples and among all stable partnerships is correspondingly lower. Furthermore, Celum *et al*.'s 29% outside-infection share is based on data from a drug trial whose participants were recruited rather than sampled. If one can assume that the biological activity of a drug is similar among most people, then trials using recruited participants can produce externally valid results about the drug's effectiveness. The outside-infection share, however, is determined by both biological (per-act transmission rate) and behavioural (concurrency) factors. Estimates of the prevalence of behaviour in a population require a representative sample of the population. Epstein and Morris cannot assume that the outside-infection share in a representative sample of the SSA population is the same 29% that Celum *et al*.'s study found among recruited participants, who are not necessarily representative of the population of SSA.

The outside-infection share is affected by more than changes in transmission rates in sexual exposures or changes in patterns of concurrency. The outside-infection share could rise because of an increase in non-sexual transmission from, for example, scale-up in male circumcision without sufficient precautions to prevent iatrogenic transmission. It could also change as an epidemic matures. In early stages of an epidemic, most incident infections come from outside the couple, but as the epidemic matures, transmission increasingly occurs within stable couples even if rates of concurrency are stable.

In sum, the outside-infection share varies for reasons other than concurrency and thus is not a reliable index of concurrency's impact on HIV epidemics.
